# Systematic analysis of gut microbiota in pregnant women and its correlations with individual heterogeneity

**DOI:** 10.1038/s41522-020-00142-y

**Published:** 2020-09-11

**Authors:** Hongling Yang, Ruochun Guo, Shaochuan Li, Fang Liang, Cheng Tian, Xueqin Zhao, Yan Long, Fei Liu, Min Jiang, Yu Zhang, Jun Ma, Mengni Peng, Siyi Zhang, Weitao Ye, Qiangsheng Gan, Fangling Zeng, Shanliang Mao, Qihua Liang, Xiaodong Ma, Mengru Han, Fei Gao, Rentao Yang, Cheng Zhang, Lulu Xiao, Junjie Qin, Shenghui Li, Chunyan Zhu

**Affiliations:** 1 Department of Laboratory, Guangzhou Women and Children’s Medical Center, Guangzhou Medical University, 510623 Guangzhou, China; 2 Promegene Institute, 518110 Shenzhen, China; 3 School of Public Health, Guangzhou Medical University, 511436 Guangzhou, China; 4 Department of Gynaecology and Obstetrics, Guangzhou Women and Children’s Medical Center, Guangzhou Medical University, 510623 Guangzhou, China; 5 Loudi Health Center for Women and Children, 417000 Loudi, China; 6 Department of Neurology, National Key Clinical Department and Key Discipline of Neurology, The First Affiliated Hospital, Sun Yat-sen University, 510275 Guangzhou, China; 7 Nanfang Hospital, Southern Medical University, 510515 Guangzhou, China

**Keywords:** Microbiome, Policy and public health in microbiology

## Abstract

The woman’s gut microbiota during pregnancy may support nutrient acquisition, is associated with diseases, and has been linked to infant health. However, there is limited information on gut microbial characteristics and dependence in pregnant women. In this study, we provide a comprehensive overview of the gut microbial characteristics of 1479 pregnant women using 16S rRNA gene sequencing of fecal samples. We identify a core microbiota of pregnant women, which displays a similar overall structure to that of age-matched nonpregnant women. Our data show that the gestational age-associated variation in the gut microbiota, from the ninth week of gestation to antepartum, is relatively limited. Building upon rich metadata, we reveal a set of exogenous and intrinsic host factors that are highly correlated with the variation in gut microbial community composition and function. These microbiota covariates are concentrated in basic host properties (e.g., age and residency status) and blood clinical parameters, suggesting that individual heterogeneity is the major force shaping the gut microbiome during pregnancy. Moreover, we identify microbial and functional markers that are associated with age, pre-pregnancy body mass index, residency status, and pre-pregnancy and gestational diseases. The gut microbiota during pregnancy is also different between women with high or low gestational weight gain. Our study demonstrates the structure, gestational age-associated variation, and associations with host factors of the gut microbiota during pregnancy and strengthens the understanding of microbe–host interactions. The results from this study offer new materials and prospects for gut microbiome research in clinical and diagnostic fields.

## Introduction

The gut microbiota has been associated with wellness and disease^1^. The woman’s gut microbiota is essential during pregnancy^2,3^ due to its crucial roles in nutrient acquisition^4^, immune remodeling^5^, and protection against infection^6^. In recent years, many studies have investigated the temporal variation in the gut microbiota in pregnant women and revealed a dramatically altered structure of the gut microbiota at different stages of pregnancy^7,8^ or, conversely, a relatively stable gut microbial profile during pregnancy^9^. The inconsistent observations of these studies could be due to the complexity and mutability of the gut microbiota and the limited sample sizes. In addition, a range of parameters in women, including their physical condition before and during pregnancy (e.g., pre-pregnancy weight, hormone levels)^10,11^ and psychological (e.g., stress)^12^ and environmental factors (e.g., dietary habits)^13^, affect their gut microbiota over the course of pregnancy. In turn, alterations in the gut microbiota may have subsequent impacts on the occurrence of gestational diseases^14,15^, fetal status, pregnancy outcomes^16^, and even the immune development of the offspring^17,18^. A series of gut microbes have been identified as participators in these processes. For example, an anti-inflammatory commensal *Faecalibacterium* is negatively correlated with gut permeability during pregnancy^19^, decreased in women with gestational diabetes mellitus (GDM)^20^, and involved in infant gut microbial maturation^21^. Based on the current status of research, however, these findings have not always been validated by related studies^15,18^. Therefore, it is still necessary to extensively investigate the dynamics of the gut microbiota during normal pregnancies and to quantitatively assess bacterial-level variations resulting in host heterogeneity, especially in a large cohort-based study.

In the current study, we performed a population-level investigation of the gut microbiotas of 1479 pregnant women of Chinese origin, with a broad range of gestational ages, from the 9th week of gestation to antepartum (>36th week). We used 16S rRNA gene sequencing technology and bioinformatic analyses to identify microbial diversity and compositional and functional characteristics across pregnancy. Integrative analyses of 132 host parameters were also performed to investigate microbe–host associations.

## Results

### Description of the study cohort

The study cohort consisted of 1479 pregnant women with a mean age of 30.6 ± 4.3 years (mean ± SD, range 18–45 years). All participants were recruited from the Guangzhou Women and Children’s Medical Center in Guangzhou, a large modern city located in Guangdong Province in South China. The exclusion criteria included participants who had taken antibiotic treatment or probiotic supplements in the 4 weeks prior to sample collection, strict vegetarians, and individuals with alcoholism or with other unusual dietary habits. The basic characteristics of all participants are summarized in Table 1. The cohort was comprised of the predominant gestational stages from the first pregnancy examination (usually the ninth gestational week or later) to the antepartum period. Notably, to increase the representativeness of the whole population, pregnant women were randomly selected in the recruiting process, which led to a uniform distribution between the 13th and 36th gestational week (per 4-week stage, *n* = 204 ± 95) but few participants in the first trimester (9th–12th week, *n* = 50) and at the antenatal period (≥37th week, *n* = 30).

**Table 1:** Basic characteristics of the 1479 pregnant women in this study.

Gest. age stages (# of samples)		Descriptive measurement of pregnant women		Biochemistry index at sampling		Disease information (% of individuals)	
9–12 weeks	52	Age (years)	30.6 ± 4.2	ALT (mmol/L)	17.7 ± 17.3	GDM	18%
13–16 weeks	303	Residency status (% native)	72.5	AST (mmol/L)	18.2 ± 6.8	Gest. hypertension	1.8%
17–20 weeks	298	Pre-pregnancy weight (kg)	52.5 ± 7.2	GLU (mmol/L)	4.3 ± 0.5	Gest. infection	4.3%
21–24 weeks	207	Pre-pregnancy BMI (kg/m^2^)	20.7 ± 2.7	HbA1c (%)	5.2 ± 0.3	Thalassemia	5.8%
25–28 weeks	171	SBP (mmHg)	107 ± 12	UA (mmol/L)	252 ± 62	Hepatopathy	7.3%
29–32 weeks	256	DBP (mmHg)	60 ± 8	Urea (mmol/L)	2.7 ± 0.6	Hypothyroidism	5.1%
33–36 weeks	162	Weight at delivery (kg)	65.7 ± 7.9	γGT (mmol/L)	12 ± 6	Hysteromyoma	3.2%
≥37 weeks	30	Gest. age at delivery (days)	275 ± 10	A/G	1.3 ± 2	Breast disease	0.7%
		Preterm birth (% individuals)	4.7	PT (s)	12.3 ± 0.5	Scar uterus	16.6%
		GWG (kg)	13.2 ± 4.5	INR	0.93 ± 0.05	Ovarian disease	1.3%
		GLU 0 min (mmol/L)^a^	4.4 ± 0.3	TBIL (μmol/L)	7.3 ± 2.4		
		GLU 60 min (mmol/L)	7.8 ± 1.6	TBA (μmol/L)	1.9 ± 2		
		GLU 120 min (mmol/L)	6.9 ± 1.4	RBC (blood)	4 ± 0.4		
				WBC (blood)	10 ± 2.2		
				WBC (urine)	33.6 ± 79.2		
				pH (urine)	6.4 ± 0.6		

The data are presented as the mean ± SD.

*BMI* body mass index, *SBP* systolic blood pressure, *DBP* diastolic blood pressure, *GWG* gestational weight gain, *GLU* blood glucose, *ALT* alanine transaminase, *AST* glutamic oxaloacetic transaminase, *UA* uric acid, *A/G* albumin/globulin, *PT* prothrombin time, *INR* international normalized ratio, *TBIL* total bilirubin, *TBA* total bile acids, *RBC* red blood cells, *WBC* white blood cells.

^a^The blood glucose levels were detected by a 75 g oral glucose tolerance test (OGTT) at the 23th–29th gestational week for the diagnosis of GDM.

To investigate the host variables that are associated with the microbiota during pregnancy, we analyzed 132 exogenous and intrinsic host factors of the participants, including 22 intrinsic host properties, 63 biomedical indices (52 blood parameters and 11 urine parameters), 20 self-reported or clinically diagnosed diseases, 7 hepatitis virus infection statuses, and 20 parameters of pregnancy outcome and neonatal information (a full list of these factors is shown in Supplementary Data 1). A total of 72.5% of the women were native residents, and the others were immigrants who had lived in Guangzhou for at least 1 year. Before pregnancy, 14% of the women were lean (body mass index [BMI] < 18 kg/m^2^) and 7% of the women were overweight/obese (BMI ≥ 25 kg/m^2^). A total of 36.8% (934/1479) of the women had at least one disease before or during pregnancy; in particular, 18% of the women had GDM.

### Gut microbiome landscape during pregnancy

The gut microbiotas of 1479 fecal samples of pregnant women were characterized by sequencing the V4 variable region of the bacterial 16S rRNA gene, generating a total of 75.8 million high-quality sequences (51,251 ± 12,403 sequences per sample; minimum 27,883). A total of 7701 operational taxonomic units (OTUs) were identified and taxonomically annotated based on an open source, universal microbiome bioinformatics platform (QIIME2)^22^. Of these OTUs, 32.5% could be robustly annotated into a known species (representing 62.9% of the total sequences), and 86% could be annotated into a genus or a family (representing 96.9% of the total sequences).

**Fig. 1:**
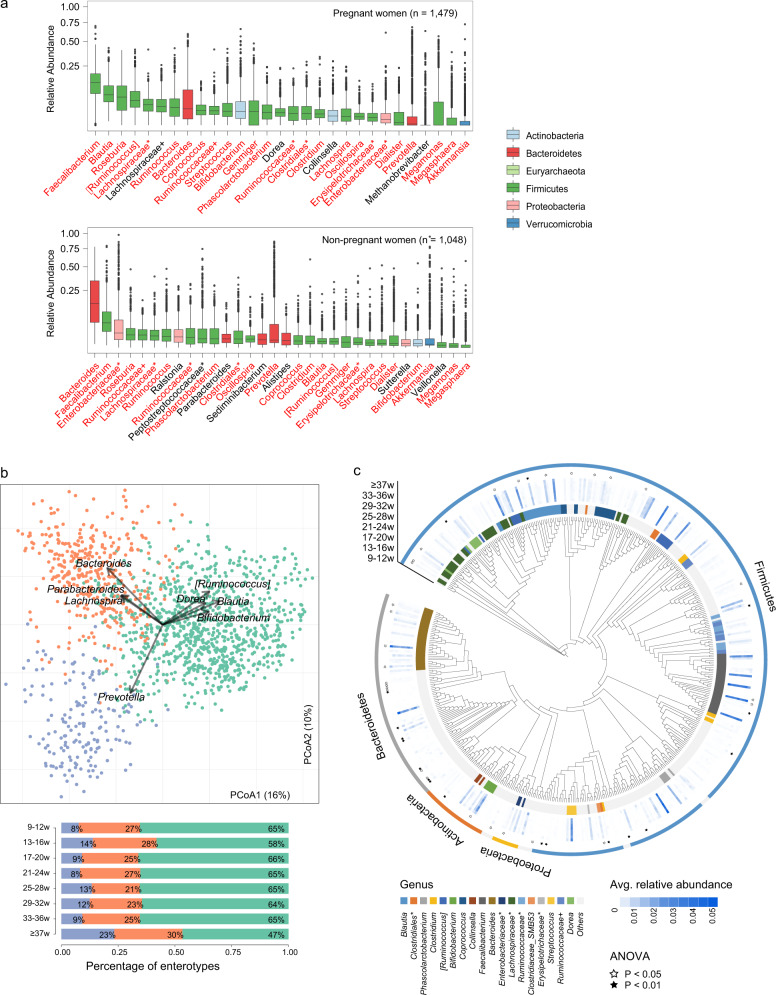
Gut microbiome landscape during pregnancy. **a** Comparison of core genera (mean relative abundance >0.5%) between the pregnant women and the GGMP nonpregnant women. Twenty-five genera marked in red were dominant genera of both the pregnant women and GGMP nonpregnant women. **b** The enterotype analysis based on Jensen–Shannon divergence of the genus profile. The scatter plot shows that the pregnant women are split into three enterotypes, and the arrows indicate the top eight contributors to compositional variation. The bar chart shows the percentage of enterotype at each stage of pregnancy. **c** The overall landscape of all fecal samples from the pregnant women based on 489 core OTUs. Colored blocks of the circle nearest phylogenetic tree indicate genera and of the outermost circle indicate phyla. The heat map shows the mean relative abundance of each OTU in different stages of pregnancy. Blank asterisks indicate significantly at *P* ≤ 0.05 between the eight groups, and black asterisks indicate significantly at *P* ≤ 0.01 (one-way ANOVA test).

Comprehensively, we noted that the gut microbiota of pregnant women was dominated by two major bacterial phyla, Firmicutes (accounting for 78.8% of total sequences) and Bacteroidetes (11.9%) (Supplementary Fig. 1), followed by Actinobacteria (5.6%), Proteobacteria (1.8%), Verrucomicrobia (0.7%), and Euryarchaeota (an archaeal phylum, 0.6%). This compositional pattern of bacterial phyla is generally seen in the human gut microbiota and is widely observed in normal nonpregnant adult populations^23,24^, despite the occasional perturbance of the low-abundance phyla^25^. To test whether the compositional pattern of pregnant women is stable at lower taxonomic levels, we then compared our cohort to the Guangdong Gut Microbiome Project (GGMP) cohort^26^ (consisting of 7009 healthy adults enrolled from 14 districts in Guangdong Province) at the bacterial genus level. In the GGMP cohort, there were 1048 nonpregnant women (age 18–44 years) included in the current comparison. The pregnant women yielded 29 dominant genera with a mean relative abundance >0.5%, and 25 (86.2%) of these genera were also presented among the 32 dominant genera in the GGMP nonpregnant women (Fig. 1a and Supplementary Data 2 and 3). These results highlight the compositional similarities at lower taxonomic levels between nonpregnant and pregnant women, despite differences in sample preparation and DNA extraction approaches.

The primary structure of the human gut microbiome is described by enterotype^27^. As expected, pregnant women exhibited consistent enterotype patterns with normal adults, which were clearly driven by the abundance of several dominant genera, such as *Bacteroides*, *Prevotella*, and *Ruminococcus* (Fig. 1b). This enterotype composition was relatively stable across all gestational stages (*P* > 0.05, analysis of variance [ANOVA] test), with a slight reduction in *Ruminococcus*-type in the last stage of pregnancy.

To provide a systematic view of the human gut microbiota across pregnancy, we portrayed the overall landscape of all fecal samples based on 489 core OTUs (mean relative abundance across all samples, >0.01%; Fig. 1c and Supplementary Data 4). A total of 345 (70.6%) OTUs were members of Firmicutes, while 87 (17.8%) OTUs were members of Bacteroidetes; this finding was in agreement with previous reports showing the highest taxonomic diversity of these two phyla in the human gut^23^. These 489 OTUs comprised 95.2% of the total abundance (for each individual, range from 95% to 95.4%) during different gestational stages, representing a relatively stable “core microbiota” across pregnancy. Notably, only 1.2% (6/489) of these OTUs were significantly different (*q* ≤ 0.05, ANOVA test) when comparing different gestational stages (Supplementary Data 4), including 1 Bacteroidetes OTU that showed increased abundance in the first trimester (9th–12th week) and the antepartum month (≥37 weeks) and 5 Firmicutes OTUs that showed various trends during pregnancy (Supplementary Fig. 2). Increasing the mean relative abundance cutoff to 0.1% reduced the core microbiota to 129 OTUs, representing 82.2–84.1% of the total abundance at different gestational stages (Supplementary Data 4), while only 1 OTU (F0112, assigned to unclassified Peptostreptococcaceae) was associated with gestational stage.

In terms of microbial function, the pregnant women’s functional microbiome was composed of 5790 Kyoto Encyclopedia of Genes and Genomes (KEGG) orthologs (KOs), which were further integrated into 525 modules (representing 67.4% of total KO abundance). Of these, 1517 KOs and 272 modules were core functions with a mean relative abundance cutoff of 0.01%, representing 94.3% and 66.9% of the total abundance, respectively (Supplementary Data 5). The pregnant women’s microbial functional composition revealed high coherence compared with their phylogenetic composition (Supplementary Fig. 3a, Procrustes *M*^2^ = 0.661, *P* ≤ 0.001) and was also markedly separated among individuals with three enterotypes (Supplementary Fig. 3b). In addition, similar to the phylogenetic composition, the core functional microbiome of the pregnant women was very similar to that of nonpregnant women (Supplementary Fig. 3c).

### Evidence of gut microbial alterations associated with gestational age

To investigate differences in the gut microbiota at different stages of pregnancy across their subjects, we first assessed the microbial *α* (within-sample) and *β* (between-sample) diversity in terms of the OTU profiles. No significant differences in *α* diversity were detected during the pregnancy period (ANOVA *P* > 0.05 for all four estimators of *α* diversity, Supplementary Fig. 4); likewise, the *β* diversity between different gestational stages did not differ significantly (*P* > 0.05 for all 4 estimators).

We then tested whether gestational age was associated with the community structure over the entire cohort. Gestational age accounted for 0.12% (*adonis* *q* = 0.026) and 0.14% (*adonis* *q* = 0.049) of the gut microbiota variance at the OTU and genus levels, respectively. This effect size was significant but quite smaller than that of the enterotype stratification and was relatively smaller than those of other host parameters (see sections below). Unconstrained redundancy analysis based on Bray–Curtis distance between microbial genera did not show any trends across gestational age (Supplementary Fig. 5a), while gestational age-constrained redundancy analysis captured visible effects on the overall gut microbiota that approached significance (*P* = 0.06; explainable proportion of variance in the top two constrained axes, 0.2% and 0.1%, respectively; Supplementary Fig. 5b). Several genera, including *Ruminococcus*, *Dialister*, *Bifidobacterium*, *Blautia*, and *Lachnospiraceae-[Ruminococcus]* (abbreviated to *[Ruminococcus]* in later sections), represented the major contributors to the gestational age-constrained axes (Supplementary Fig. 5b); the abundance of these genera changed throughout pregnancy. These data together suggest that the influence of gestational age on women’s gut microbiota is limited, but considerable.

**Fig. 2:**
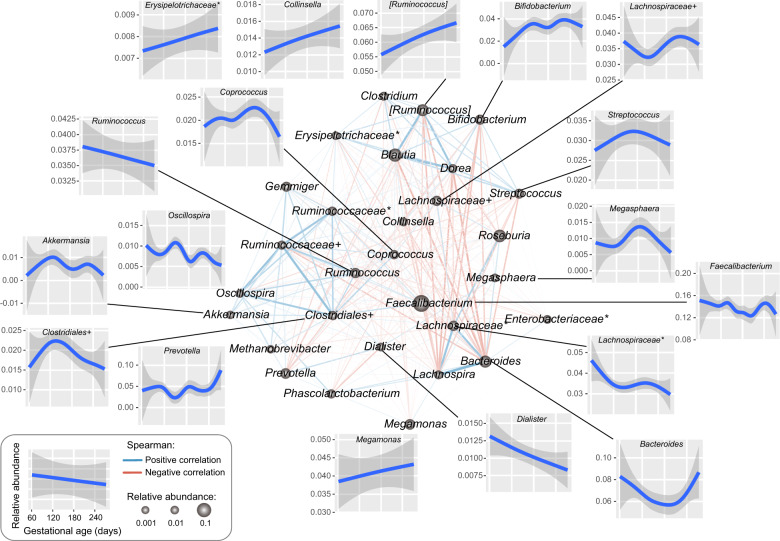
Alterations of the core genera in pregnant women across pregnant stages. The network consisted of significant positive (blue) and negative (red) correlations between 29 core genera with a mean relative abundance >0.5% (Spearman’s rank correlation coefficient test, *q* ≤ 0.05). Line width is proportional to the degree of correlation, and point size is proportional to the mean relative abundance of core genera in all samples. Outer graphs show the response pattern for the core genera across pregnant stages, and a smooth curve is formed based on the relative abundance of each genus and gestational days of the sample using the function *geom_smooth* with the default parameters in the R platform.

We produced the correlation network of 29 dominant genera based on the relative abundance in subjects at different stages of pregnancy (Fig. 2 and Supplementary Data 2). These genera were tightly correlated among all individuals and even in subsets of individuals at each gestational stage (Supplementary Fig. 6), indicating that a strongly shared relationship among the gut microbes existed throughout pregnancy. Four genera, *[Ruminococcus]*, *Collinsella*, *Megamonas*, and *unclassified-Erysipelotrichaceae*, increased continuously with gestational age, whereas *Ruminococcus*, *Dialister*, and *unclassified-Lachnospiraceae* decreased continuously. Parallelly, *Streptococcus*, *Megasphaera*, *unclassified-Clostridiales*, and *Bacteroides* seemed to be the most common taxa in midtrimester. *Streptococcus* and *Megasphaera* were enriched at 21–28 weeks of pregnancy, *unclassified-Clostridiales* was enriched at 17–24 weeks, and *Bacteroides* was reduced at 21–28 weeks. The combination of these 11 gestational-age-associated genera revealed a moderate performance in predicting gestational age (Supplementary Fig. 7, Spearman’s *ρ* = 0.14, *P* = 7.4e−6), suggesting that these genera may be potential microbial markers.

### Host properties and blood parameters are more strongly associated with microbial community composition than gestational age

**Fig. 3:**
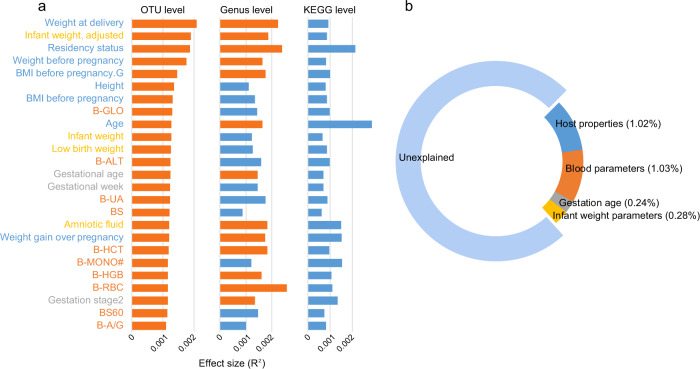
The effect sizes of host parameters on microbial community variation. **a** The effect sizes of 25 host parameters that are significantly correlated with microbial community variation at the OTU profiles. The effect sizes of these parameters are also shown at the genus KO levels. Red bars indicate significance at *adonis* *q* ≤ 0.05, and blue bars indicate no significant differences. Parameters are divided into four categories and are colored based on their types (defined in Supplementary Data 1). **b** Combined effect sizes of four categories on the overall microbial community variation at the OTU level.

We identified 25 host parameters (19% of all collected parameters) that significantly correlated with overall microbial community variation at the OTU level (minimum *R*^2^ = 0.11%, *q* ≤ 0.05; Fig. 3a and Supplementary Data 6). These parameters included 8 basic host properties, 10 blood clinical parameters, 4 parameters associated with neonate and pregnancy outcomes, and 3 parameters of gestational age. The weight-associated parameters of both pregnant women (weight at delivery, pre-pregnancy weight, pre-pregnancy body mass index [PBMI], and gestational weight gain [GWG]) and their infants (adjusted infant weight, infant weight, and low birth weight) were the most significant. Importantly, all 8 host properties, including residency status, height, age, and the 5 maternal weight parameters, explained a combination of 1.02% gut microbial variation (Fig. 3b), while the combination of 10 blood parameters explained 1.03% of the variation. As a comparison, the 3 gestational age parameters explained a total of only 0.24% of the variation. Unsurprisingly, analysis of individuals at each gestational stage also revealed that the explicable variations in host properties and blood parameters were 3–5-fold larger than the variations in gestational age (Supplementary Fig. 8). Thus these results indicated that, other than gestational age, host heterogeneity is associated with the gut microbiota throughout pregnancy.

The half (52%) of these parameters were also validated at the genus level of microbial composition at *q* ≤ 0.05; however, only 6 parameters were correlated with the functional profiles at a milder condition of *q* ≤ 0.3 (*P* ≤ 0.05, Fig. 3a). Noticeably, the effect sizes of the pregnant women’s age (*R*^2^ = 0.29%, *adonis* *P* = 0.002, *q* = 0.089) and residency status (*R*^2^ = 0.21%, *adonis* *P* = 0.001, *q* = 0.089) were most prominent in the functional profiles, confirming that the effect of host heterogeneity on gut microbial functions is notable. Likewise, the combination of 6 function-correlated parameters explained 1.46% of the gut functional variation, similar to the scale of the variation at the OTU and genus levels.

**Fig. 4:**
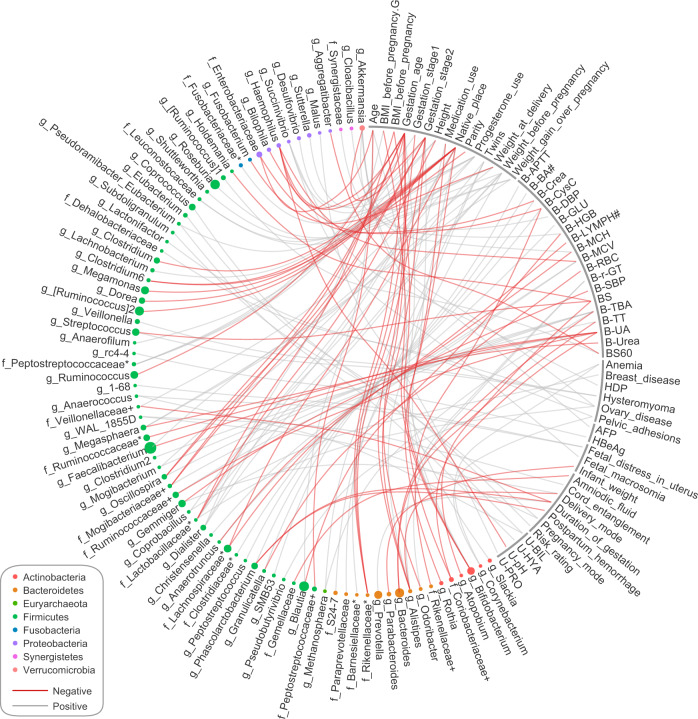
A correlation network between bacterial genera and host parameters. The network shows all significant correlations between genera and host parameters in the pregnant women (Spearman’s rank correlation coefficient test, *q* ≤ 0.05). The black lines and colored nodes on the ring represent host parameters and genera, respectively. Node size is proportional to the mean relative abundance of genera in all samples, and node color indicates phylum. Gray lines indicate positive correlation, and red lines indicate negative correlation.

We performed multivariate analysis by linear models (MaAsLin)^28^, which allowed us to capture the correlations between each parameter and microbial genus by deconfounding the effects of other unrelated parameters. We identified 137 significant inter-associations (*q* ≤ 0.20, corresponding to *P* < 0.008) between the host parameters and microbial genera (Fig. 4 and Supplementary Data 7). The basic host properties (residency status, age, height, and weight parameters; number of associations: *n* = 35) and blood parameters (*n* = 38) composed the major percentage of these associations, followed by the gestational age parameters (*n* = 19). One parameter, the pregnant women’s residency status, correlated with the largest number of genera including several important members of Lachnospiraceae (e.g., *[Ruminococcus]*, *Dorea*). Our population-level data validated the previous observation of a potential correlation between host parameters and gestational gut microbial composition in small datasets (see “Discussion”) and found novel associations such as the negative correlation between PBMI and *Bifidobacterium* (Supplementary Fig. 9).

### Women’s age, PBMI, and residency status describe the gestational microbial composition and function

To identify the host property-associated OTUs that could play an exact role in shaping the gestational gut microbiome, we compared the gut microbial community compositions and functions of pregnant women between the top 75% and bottom 25% of individuals in terms of age (older group: ≥34 years, *n* = 369; younger group: ≤27 years, *n* = 359) and PBMI (obese group: >22.1, *n* = 368; lean group: <18.8, *n* = 368), as well as compared their types of residency (native residents, *n* = 966; immigrants, *n* = 407). Among these groups, the older and obese women, as well as the younger and lean women, were statistically overlapped (chi-squared test *χ*^2^ = 68, *P* < 2.2e–16), in agreement with the previous population study showing that the BMI of reproductive age women is continuing to increase with age^29^.

#### Age

**Fig. 5:**
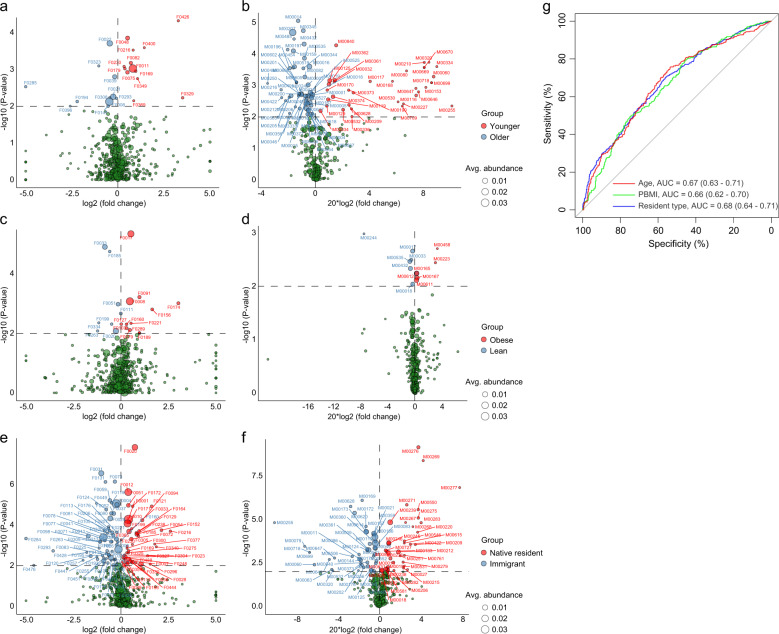
Comparison of the gut microbial community compositions and functions among different groups. **a**–**f** Comparison of the OTUs and KEGG modules (at level D) between younger and older (**a**, **b**), lean and obese (**c**, **d**), and native residents and immigrants (**e**, **f**) in the gut microbiota of pregnant women. Colored circles indicate OTUs or modules that were enriched in the corresponding group, and the sizes of the circles indicate their mean relative abundances. **g** Receiver operating curves for age, PBMI, and residency status. Random forest models based on significantly different OTUs were assessed with leave-one-out cross-validation.

Twenty-four core OTUs (4.9%) were associated with age, of which 11 were enriched in older women and 13 were enriched in younger women (Wilcoxon rank-sum test *P* ≤ 0.01, corresponding to *q* < 0.173; Fig. 5a and Supplementary Data 8). The older-enriched OTUs belonged to Ruminococcaceae (F0039 and F0396), *Blautia* (F0021 and F0022), *Dialister* (F0194 and F0285), Mogibacteriaceae (F0308), *Oscillospira* (F0147), Clostridiales (F0323), *Faecalibacterium prausnitzii* (F0008), and *Prevotella copri* (F0293), while the younger-enriched OTUs included *Veillonella* (F0075, F0169 and F0400), *P. copri* (F0011 and F0329), Enterobacteriaceae (F0048), *Haemophilus parainfluenzae* (F0082), Bacteroidales_S24-7 (F0349), *Clostridium perfringens* (F0216), *Dialister* (F0369), *Lactobacillus* (F0426), *Rothia mucilaginosa* (F0230), and *Turicibacter* (F0179). The negative correlation of Enterobacteriaceae with women’s age was in agreement with the observation at the genus level (Fig. 4). Of note, two older-enriched OTUs, F0008 (*F. prausnitzii*) and F0021/F0022 (*Blautia obeum*) and one younger-enriched OTU F0011 (*P. copri*), were dominated in their abundance (>1%) in the women’s gut microbiota (Supplementary Fig. 10); these OTUs may be signature taxa for pregnant women’s age. Functionally, 89 core KEGG modules were age associated (*P* ≤ 0.01, corresponding to *q* < 0.011; Fig. 5b and Supplementary Data 8), including 35 that were enriched in older women and 54 that were enriched in younger women.

#### Pre-pregnancy body mass index

Twenty core OTUs (4.1%) were associated with PBMI, including 12 that were enriched in obese women and 8 that were enriched in lean women (Wilcoxon rank-sum test *P* ≤ 0.01, corresponding to *q* < 0.166; Fig. 5c and Supplementary Data 9). The obese-enriched OTUs were assigned to Lachnospiraceae (F0091, F0127 and F0221), Ruminococcaceae (F0079 and F0289), Clostridiales (F0156, F0160 and F0174), *F. prausnitzii* (F0008), *Roseburia faecis* (F0017), *Adlercreutzia* (F0103), and *Anaerostipes* (F0189), while the lean-enriched OTUs included *Oscillospira* (F0199 and F0263), Ruminococcaceae (F0111 and F0334), *[Ruminococcus] gnavus* (F0020), *Clostridium ramosum* (F0185), *Bifidobacterium* (F0033), and *Streptococcus* (F0051). The negative correlation between *Bifidobacterium* and PBMI was consistent with the observation at the genus level. Three dominant OTUs with a minimum mean abundance >1% in all individuals were F0008 (*F. prausnitzii*), F0017 (*R. faeces*), and F0020 (*[Ruminococcus] gnavus*) (Supplementary Fig. 11). Functionally, 12 core KEGG modules were PBMI associated (*P* ≤ 0.01, corresponding to *q* < 0.128; Fig. 5d and Supplementary Data 9), including 6 that were enriched in lean women and 6 that were enriched in obese women.

#### Residency status

A total of 103 core OTUs (21.1%) were associated with women’s type of residency, including 43 that were enriched in native women and 60 that were enriched in immigrants (Wilcoxon rank-sum test *P* ≤ 0.01, corresponding to *q* < 0.023; Fig. 5e and Supplementary Data 10). Several native-enriched OTUs, including *[Ruminococcus] gnavus* (F0020), *Megamonas* (F0001), *Blautia* (F0012), Lachnospiraceae (F0007), and *Clostridium* (F0023), as well as several immigrant-enriched OTUs, including *F. prausnitzii* (F0004), *Gemmiger formicilis* (F0006), *P. copri* (F0011 and F0031) and *Ruminococcus bromii* (F0025), were of the dominant taxa (>1%) in the women’s gut microbiota. Functionally, 81 core KEGG modules were associated with residency status (*P* ≤ 0.001, corresponding to *q* < 0.01; Fig. 5f and Supplementary Data 10), including 38 that were enriched in native individuals and 45 that were enriched in immigrants.

Using the random forest model, the 24 age-associated OTUs achieved an area under the curve (AUC) of 0.67 for the classification of older and younger women in our cohort (Fig. 5g), and the 20 PBMI-associated OTUs and 103 residency-status-associated OTUs achieved a similar AUC for distinguishing obese vs. lean individuals and native residents vs. immigrants, respectively.

### The gut microbiota composition is different in women with high or low GWG

**Fig. 6:**
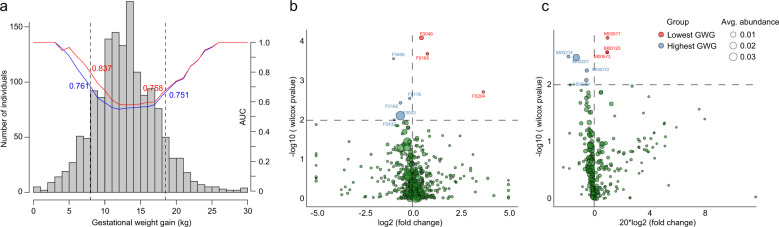
Comparison of the gut microbial community compositions and functions between low and high GWG cohorts. **a** Area under the ROC curve (AUC) for the GWG group. The blue line indicates the random forest model based on all OTUs, and the red line indicates the random forest model with added PBMI compared with the former model. Each AUC was calculated based on two groups of individuals who were higher or lower than a certain GWG value (e.g., one was <5 GWG, the other was >5 GWG value). The bar plot shows the distribution of GWG values in all samples. **b**, **c** Comparison of the OTUs and KEGG modules between the pregnant women with the lowest and highest GWG.

Women’s GWG is an important indicator for estimating nutrient absorption during pregnancy^30^. GWG was positively associated with two important short chain fatty acid (SCFA) producers^31,32^, *Faecalibacterium* and *Roseburia* (Fig. 4), in the human gut microbiota; this finding is in agreement with previous studies which showed that SCFAs are enhanced in the context of weight gain^33^. We tried to use the relative abundance of all OTUs to train a random forest model for the identification of extreme GWG individuals and obtained an AUC of 0.76 for distinguishing women with the lowest 10% GWG (132 individuals with GWG ≤7.9 kg) and an AUC of 0.75 for women with the highest 10% GWG (136 individuals with GWG ≥18.6 kg; Fig. 6a). In addition, random forest regression based on continuous GWG also revealed high consistency between the gut microbiota-predicted value and the measured GWG of all pregnant women (Supplementary Data 11), especially for women at the gestational stages of 13–16 weeks (Spearman’s *ρ* = 0.16, *q* = 0.017) and 29–32 weeks (Spearman’s *ρ* = 0.24, *q* < 0.001). These results suggested that the gut microbial composition may be a good indicator for the weight status of pregnant women. Noticeably, adding the PBMI to the regression model increased its predictive power (Supplementary Data 11), particularly PBMI was more efficient for distinguishing low GWG women (AUC = 0.84; Fig. 6a).

We then compared the gut microbial community compositions and functions of pregnant women between the women in the top 75% (GWG ≥16.5 kg, *n* = 324) and the bottom 25% (GWG ≤10 kg, *n* = 352) of GWG. Only 8 OTUs were associated with GWG at *P* ≤ 0.01 (corresponding to *q* < 0.366; Fig. 6b and Supplementary Data 12). The lowest-GWG-individual-enriched OTUs belonged to *Bacteroides uniformis* (F0049), *Dialister* (F0204), and *Clostridium* (F0165), while the highest-GWG-individual-enriched OTUs were Clostridiales (F0160 and F0176), *R. faecis* (F0003), *Paraprevotella* (F0432), and Lachnospiraceae (F0406). Noticeably, lowest-GWG-individual-enriched OTU F0049 and the highest-GWG-individual-enriched OTU F0003 were also determined as important features in the random forest regression model based on continuous GWG mentioned above (Supplementary Data 13). Functionally, 7 core KEGG modules were GWG associated (*P* ≤ 0.01, corresponding to *q* < 0.168; Fig. 6c), including 3 that were enriched in the lowest GWG women and 4 that were enriched in the highest GWG women.

### Pre-pregnancy and gestational diseases correlate with gestational gut microbiota

Pre-pregnancy and gestational diseases are clinically significant for maternal and fetal health during pregnancy^34^; however, in our cohort, the disease-involved parameters explained only minor variations (all <0.2%, Supplementary Data 6) in the gut microbiome in our dataset, mostly because of the fact that the women recruited for the study were relatively healthy adults and did not have certain severe diseases (see “Methods”). To investigate the detailed influence of the gestational gut microbiota on women’s health, three pre-pregnancy diseases (hepatopathy, hypothyroidism, and thalassemia) and gestational GDM with >5% morbidity in our cohort were analyzed.

#### Gestational diabetes mellitus

**Fig. 7:**
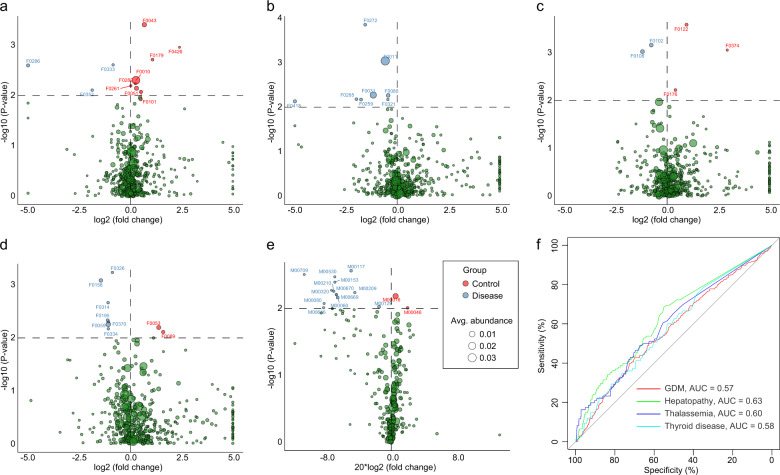
Comparison of the gut microbial community compositions and functions between the disease and non-disease groups. **a**–**d** Comparison of the OTUs between the disease and non-disease groups for GDM, hepatopathy, thalassemia, and thyroid disease. **e** Comparison of KEGG modules between patients with hypothyroidism and people without hypothyroidism. **f** Receiver operating curves for GDM, hepatopathy, thalassemia, and thyroid disease. Random forest models based on significantly different OTUs were assessed with leave-one-out cross-validation.

Eleven core OTUs (2.2%) were associated with GDM, including 3 that were enriched in GDM women and 8 that were enriched in non-GDM women (Wilcoxon rank-sum test *P* ≤ 0.01, corresponding to *q* < 0.362; Fig. 7a and Supplementary Data 14). The GDM-enriched OTUs were assigned to *B. uniformis* (F0352), *Methanobrevibacter* (F0286), and *Blautia* (F0333), while the GDM-depleted OTUs included *Streptococcus* (F0010 and F0051), *Lactobacillus* (F0426), *Turicibacter* (F0179), Peptostreptococcaceae (F0043), *Lactococcus* (F0282), Ruminococcaceae (F0101), and Lachnospiraceae (F0261). The *Streptococcus* genus was the signature taxa that was lower in GDM patients (Supplementary Fig. 12).

#### Hepatopathy

Eight core OTUs (1.6%), including *P. copri* (F0011, F0031, and F0418), *F. prausnitzii* (F0321), *Roseburia* (F0272), Ruminococcaceae (F0080), *Dialister* (F0285), and Clostridiales (F0259), were associated with hepatopathy, all of which were enriched in women with hepatopathy (Wilcoxon rank-sum test *P* ≤ 0.01, corresponding to *q* < 0.433; Fig. 7b and Supplementary Data 14).

#### Thalassemia

Five core OTUs were identified at *P* ≤ 0.01; however, none of these OTUs passed the multiple test correction (Fig. 7c and Supplementary Data 14). Two of the potential thalassemia-associated OTUs, *R. bromii* (F0108) and *Anaerostipes* (F0102), were disease enriched, while 3 control-enriched OTUs were *Bilophila* (F0122), Streptophyta (F0374), and Clostridiales (F0176).

#### Hypothyroidism

Nine core OTUs (1.8%) were associated with hypothyroidism, including 7 that were enriched in women with the disease and 2 that were enriched in non-hypothyroidism women (Wilcoxon rank-sum test *P* ≤ 0.01, corresponding to *q* < 0.336; Fig. 7d and Supplementary Data 14). The hypothyroidism-enriched OTUs were assigned to *Coprococcus* (F0059 and F0195), *Oscillospira* (F0314), Ruminococcaceae (F0326 and F0334), Lachnospiraceae (F0370), and Clostridiales (F0156), while the control-enriched OTUs were two Bacteroides (F0053 and F0089) members.

Functionally, no significant modules were found in regard to GDM, hepatopathy, or thalassemia (Supplementary Fig. 13 and Supplementary Data 14). In contrast, 15 hypothyroidism-associated modules were identified (Wilcoxon rank-sum test *P* ≤ 0.01, corresponding to *q* < 0.09; Fig. 7e). In addition, the disease-associated OTUs achieved a relatively smaller AUC for the discrimination of corresponding diseases suffered by individuals from the cohort (AUC 0.57–0.63 for four diseases; Fig. 7f).

## Discussion

Pregnancy is a complex physiological process that involves various changes to organs, nutrition metabolism, and immune and hormonal regulation to adapt to the needs of fetal growth and development. Although it is believed that the symbiotic microbiota plays a fundamental role in regulating host metabolism and responding to the environment, there are still limited data on the ensemble characteristics of the gut microbiota during normal pregnancy. This study is a large population-level survey of the gut microbiota that included 1479 pregnant women. The gut microbiomic repertoire of pregnant women provides insights into microbial structure specificity, gestational age-associated variation, and host dependence during this critical time.

In our data, the overall gut microbial structure, characterized by enterotypes, dominant taxonomic, and functional composition, of pregnant women was similar to that of age-matched nonpregnant women. The core gut microbiota is consistent in multiple healthy cohorts^24^, but its abundance profiles are usually stratified by ethnicity and region^26,35^. Our findings extended this observation in Chinese pregnant women. Further study was proposed to comprehensively investigate the gut microbial differences between two cohorts, especially the patterns that are specifically associated with gestation.

Alterations in the gut microbiota across pregnant stages have been of special concern. The current study is in agreement with a previous longitudinal study^9^ that also demonstrated a relatively stable microbiota throughout gestation, while in challenge to other finding^36^ that the gut microbiota is dramatically altered during pregnancy. As shown in previous study^37^, the gut microbiota shows relative stability during pregnancy within subjects but high inter-individual variability, so we suggest that individual heterogeneity make comparison of the gut microbiota during gestation less noticeable. In addition, dramatic changes in gut microbial composition have been observed in pregnant women after dietary intervention^7^. The dietary patterns of pregnant women largely impact their GWG as well as infant birth weight^38,39^. In our samples, however, the stability of the gut microbiota could be partly explained by the fact that the women’s dietary patterns rarely changed during normal pregnancy. Nevertheless, our results also demonstrated that changes in other pregnancy-associated physiological processes have a limited effect on the gut microbiota.

Previous population-based reports had estimated the effect size of host factors on healthy gut microbiota and explained a considerable proportion of microbial variation based on their collected parameters^24,40^. Similarly, in the current study, we strengthened the importance of host heterogeneity on pregnant women’s gut microbiota and identified a set of parameters that were strongly correlated with the variation in gut microbiota. We also identified associations between host parameters and gut microbes. Some of these associations were previously inferred, including (1) the negative association between blood thrombin time (a coagulation indicator) and *Bacteroides*, which can be explained by *Bacteroides* (e.g., *B. fragilis*) being the main producer of an important blood-clotting factor, vitamin K2, in the human body^41,42^; and (2) the frequent associations between blood total bile acid or uric acid (UA) and gut microbial members (including *Bacteroides*, *Faecalibacterium*, and Ruminococcaceae), facilitated by the fact that many gut microbes are important participates in host bile acid and UA metabolism^43^. Intriguingly, some associations such as the negative correlation between PBMI and *Bifidobacterium* have not been previously reported. In addition to these examples, there were many correlations among the considerable unexplained variance between host parameters and the gut microbiome, requiring further study to understand their significance.

Our comprehensive data on the gut microbiome of pregnant women enabled us to identify the taxonomic and functional signatures of age, PBMI, and residency status, as well as pre-pregnant and gestational diseases in a sufficient number of samples. For example, in older pregnant women, the OTUs of *F. prausnitzii* and *B. obeum* were enriched and those of *P. copri* were diminished. An increase in *F. prausnitzii* is beneficial for gut health, mostly due to its functions in butyrate production and anti-inflammatory effects^31,44,45^, while gut *P. copri* can induce insulin resistance by producing branched-chain amino acids^46^. *Faecalibacterium* was also positively correlated with GWG in our cohort. In addition, in this cohort, only 11 OTUs and no functional modules were altered between pregnant women with GDM and normoglycemic pregnant women. Rare gut taxonomic and functional variations in GDM have also been reported in women in the third trimester of pregnancy^20^. Consistently, the enrichment of *Blautia* OTUs was identified in both the previous report and our cohort, suggesting their potential role in GDM development.

In our cohort, the large difference of pregnant women’s gut microbiota between native residents and immigrants may be contributed by numerous factors, such as lifestyle, economic level, education, and disease epidemiology. As reported in a recent study^26^, in south Chinese population, the host location was the strongest associations with gut microbiota variations comparing with other phenotypes. Our result thus suggested that the effect of host location on gut microbiota can partly be retained in immigrants. This strong “ethnic origin” effect on gut microbiota was also widely observed in European^35^ and first-generation American immigrants^47^.

The association between weight-associated parameters and the gut microbiota is of particular interest. In our cohort, several parameters, including women’s weight before pregnancy and at delivery, GWG, and infant weight parameters, were associated with the overall gut microbiota as well as some gut microbes. The correlation of some bacteria with GWG, e.g., a negative correlation between *Paraprevotella*/Lachnospiraceae and GWG, partly overlapped with the observations in previous studies^10^. Moreover, there was significant correlation between the observed and the predicted values for GWG regression models based on samples at two gestational stages (13–16 and 29–32 weeks), but not the others. This suggested a good potential of the gut microbiota for the prediction of the energy and nutrient metabolism of women at particular gestational age. Noticeably, PBMI may be necessary for building more accurate GWG prediction models based on microbiome composition.

Because our study design was limited in that each participant provided one fecal sample for analysis, we lacked longitudinal data to fully determine the dynamic variation in each pregnant woman throughout gestation. In addition, the current study does not provide any mechanistic explanation for the variation in host heterogeneity and gut microbiota.

## Methods

### Study design and sample collection

This study received approval from the Ethics Committee of Guangzhou Women and Children’s Medical Center (No. 2018030306). Written informed consent was obtained from all participants, and no financial compensation was provided. The methods were carried out in accordance with the approved guidelines. All pregnant women who visited the prenatal clinics at Guangzhou Women and Children’s Medical Center from January 2017 to September 2017 were asked to participate in our study. The recruited pregnant women were invited to fill in a questionnaire, including their basic information (age, birthplace, living area, gravidity, parity, etc.), lifestyle data, medical history, and a self-administered food frequency questionnaire. Our exclusion criteria included (1) usage of the following drugs in the previous 6 months: systemic antibiotics, corticosterone, cytokines, methotrexate or other immunotoxic drugs, hormonal contraceptives, and high dose of commercial probiotics; (2) presence of risky diseases: serious cardiovascular disease, inflammatory bowel disease, irritable bowel syndrome, and celiac disease; (3) human immunodeficiency virus infection; (4) intestinal surgery within 5 years; (5) chronic diarrhea caused by *Clostridium difficile* or an unknown agent; (6) chronic constipation; (7) unusual dietary habits such as individuals with alcoholism and strict vegetarians; and (8) conventional antibiotic treatment or probiotic supplement in the preceding 4 weeks. Fecal samples were obtained from participants by using a sterile toilet and a fecal collection bag (including three sterile fecal collection boxes and collection spoons) during the hospital stay. For each participant, 3 parallel samples were subpackaged and frozen in −80 °C freezers within 30 min of collection.

### Medical information collection

Blood and urine samples were obtained from the pregnant women on the same day as the fecal sample collection, and the blood and urine parameters were measured by standard procedures and obtained from hospital records. Except that the blood glucose parameters BS (fasting blood glucose), BS60 (1 h blood glucose), and BS120 (2 h blood glucose) were obtained by a standard 2-h 75 g oral glucose tolerance test between 23 and 29 weeks of gestation for GDM diagnosis. Based on the criteria^48^, pregnant women were diagnosed with GDM if ≥1 of the following criteria were met: BS ≥ 5.1 mmol/L, BS60 ≥ 10.0 mmol/L, or BS120 ≥ 8.5 mmol/L. Pre-pregnancy weight was based on pregnant women’s self-report at the first clinic visit (usually 9–12 weeks of gestation). Pregnant women’s body measurement index (height, prenatal weight, blood pressure, etc.), gestational disease status, hepatitis virus infection, pregnancy outcomes, and neonatal information were documented from hospital records.

### DNA extraction and sequencing

Total bacterial DNA was extracted from the fecal samples using the MOBIO PowerSoil® DNA Isolation Kit 12888-100 protocol and stored at −80 °C in Tris-EDTA buffer solution before use. We amplified the V4 region of the 16S rRNA gene using the universal 515F (5’-GTGYCAGCMGCCGCGGTAA-3’) and 806R (5’-GGACTACNVGGGTWTCTAAT-3’) primers, along with barcode sequences for each sample. Each reaction mixture contained 10 μM each of forward and reverse primers, 1000 ng of template DNA, 200 μM of dNTPs, 5 μL of 10× EasyPfu Buffer, and 2.5 units Easy Pfu DNA Polymerase in 50 μL reaction. The PCR conditions were as follows: 95 °C for 5 min followed by 30 cycles of 94 °C for 30 s, 60 °C for 30 s, and 72 °C for 40 s, and a final extension step at 72 °C for 4 min. PCR products were separated by agarose gel electrophoresis, and the expected band size for 515F–806F was approximately 300–350 bp. DNA quantification was conducted using a Quant-iT PicoGreen dsDNA Assay Kit (ThermoFisher/Invitrogen cat. no. P11496) following the manufacturer’s instructions. The amplicon library was combined in equimolar amounts and subsequently quantified (KAPA Library Quantification Kit KK4824) according to the manufacturer’s instructions. In addition, 150 bp paired-end sequencing was performed on the Illumina MiniSeq platform at Promegene Co. Ltd. (Shenzhen, China).

### Bioinformatic analyses

#### 16S rRNA sequencing analysis

16S rRNA amplicon sequences were processed using QIIME2^49^. All reads were truncated at the 150th base with a median *Q* score >20 to avoid sequencing errors at the end of the reads. Noisy sequences, chimeric sequences, and singletons in the sequence data were removed by DATA2^50^. Denoised paired-end reads were joined, setting a maximum mismatch parameter of two bases. The representative sequences (i.e., the features) were defined at 100% similar merged sequences. We used the term “operational taxonomic unit (OTU)” instead of “feature” in the whole article for convenience. Then the taxonomy of the features was identified using the classify-sklearn classification methods based on the Greengenes 13.8 database (https://data.qiime2.org/2018.11/common/gg-13-8-99-515-806-nb-classifier.qza) via the q2-feature-classifier plugin. The phylogenetic analysis was performed in QIIME2 with “qiime alignment mafft,” “qiime alignment mask,” and “qiime phylogeny fasttree” commands, based on the tutorials at https://docs.qiime2.org/2019.1/tutorials/moving-pictures/. The phylogenetic tree of the core OTUs was visualized using iTOL v4^51^. To measure the gut microbiota diversity (including alpha and beta diversity) and quantify the taxonomic composition of the samples, all samples were rarefied to an even sampling depth of 20,000 sequences.

#### Public dataset

To compare the gut microbiota of pregnant women with that of nonpregnant women, we downloaded the raw 16S rRNA gene sequencing data of the GGMP project^26^ from the European Nucleotide Archive database (accession no. PRJEB18535). Only data from nonpregnant women aged 18–44 years were included. Data were processed using the same method as mentioned above.

#### Alpha and beta diversity

Alpha and beta diversities were calculated in the QIIME2 platform with the “qiime diversity core-metrics-phylogenetic” command. The Shannon’s diversity index, observed OTUs, Faith’s phylogenetic diversity (a qualitative measure of community richness that incorporates phylogenetic relationships between the OTUs), and Pielou’s evenness were used to reflect community richness and evenness. The Jaccard distance, Bray–Curtis distance, and unweighted and weighted UniFrac distances were implemented to assess the similarity or dissimilarity between individuals.

#### Enterotype analysis

Enterotype assignment was performed based on the published tutorial (http://enterotype.embl.de/enterotypes.html)^27^. Fecal samples were delineated into three enterotypes based on their genus-level relative abundance profiles using the Jensen–Shannon divergence and reference-based alignment algorithm.

#### Functional annotation and analysis

Functional profiling of the samples was performed using the PICRUSt2 algorithm^52^. For each sample, the composition of KOs^53^ was predicted based on the functional information of the reference OTUs. KEGG module and pathway composition was generated according to the assignment of KOs at https://www.kegg.jp/.

### Statistical analyses

Statistical analyses were implemented at the R v3.4.2 platform (https://www.r-project.org/).

#### Multivariate statistics

Principal coordinate analysis (PCoA) was carried out using the *ape* package in R. In the PCoA analysis, the between-sample Bray–Curtis distance was implemented for compositional data (including OTU, genus, and KO profiles) and was calculated using the *vegdist* function in the *vegan* package. Distance-based redundancy analysis (dbRDA) was performed on OTUs and taxonomic composition profiles with the *vegan* package, based on the Bray–Curtis distance, and visualized using the R *ade4* package. Procrustes analysis was used to determine similarity between two multivariate axes (e.g., generated from the PCoA or dbRDA analysis) and was performed with the R vegan package. The Procrustes *P* value was generated based on 1000 permutations. MaAsLin^28^ analysis was performed using default parameters (http://huttenhower.sph.harvard.edu/maaslin).

#### Effect size analysis

We estimated the effect size of each variable (host parameter) on microbiome variation using the *adonis* function within the R *vegan* package. The combined effect size of multiple host parameters was also calculated using the *adonis* function.

#### Prediction model

This analysis was carried out using the *randomForest* and *pROC* packages in R. OTUs (or KOs) with significantly different abundances between groups were selected to build the random forest model (*n* trees, 2000), and the performance of the model was assessed with leave-one-out cross-validation. The AUC was calculated using the *roc* function.

#### Statistical test

All statistical tests, including Student’s *t* test, Wilcoxon rank-sum test, and one-way ANOVA test, were performed on the R platform. Spearman correlation test was assessed using the *cor.test* function with “method=spearman” parameter. The *q* value was used to evaluate the false discovery rate for correction of multiple comparisons and was calculated based on the R *fdrtool* package. For a single test, *P* ≤ 0.05 was considered statistically significant. For multiple test, unless otherwise mentioned, *q* ≤ 0.05 was considered significant. For MaAsLin analysis, the threshold *q* ≤ 0.2 was considered significant^54^. For the association analyses between gut microbial/functional compositions and host parameters (including age, PBMI, residency status, GWG, and disease status), the threshold *P* ≤ 0.01 was used for presenting the results, and simultaneously the corresponding *q*-values was shown for estimating the significant level.

### Reporting summary

Further information on experimental design is available in the Nature Research Reporting Summary linked to this article.

## Supplementary information

Supplementary Information

Description of Additional Supplementary Files

Supplementary Data 1

Supplementary Data 2

Supplementary Data 3

Supplementary Data 4

Supplementary Data 5

Supplementary Data 6

Supplementary Data 7

Supplementary Data 8

Supplementary Data 9

Supplementary Data 10

Supplementary Data 11

Supplementary Data 12

Supplementary Data 13

Supplementary Data 14

Supplementary Data 15

Supplementary Data 16

Supplementary Data 17

Reporting Summary

## Data Availability

The raw sequencing dataset analyzed in this study has been deposited in the European Bioinformatics Institute (EBI) database under the accession code PRJEB31743. The sample metadata and OTU data are provided as Supplementary Data 15–17 for reproducibility of this study. The taxonomic composition data and the statistical scripts are available from the corresponding authors upon reasonable request.
