# Supplementary material for: Systematic analysis of gut microbiota in pregnant women and its correlations with individual heterogeneity
Source: NPJ Biofilms Microbiomes. 2020 Sep 11;6:32. doi: 10.1038/s41522-020-00142-y (PMC7486914; doi:10.1038/s41522-020-00142-y)
Supplement: Supplementary file 1 — Supplementary Information [file 41522_2020_142_MOESM1_ESM.pdf]

Supplementary Information

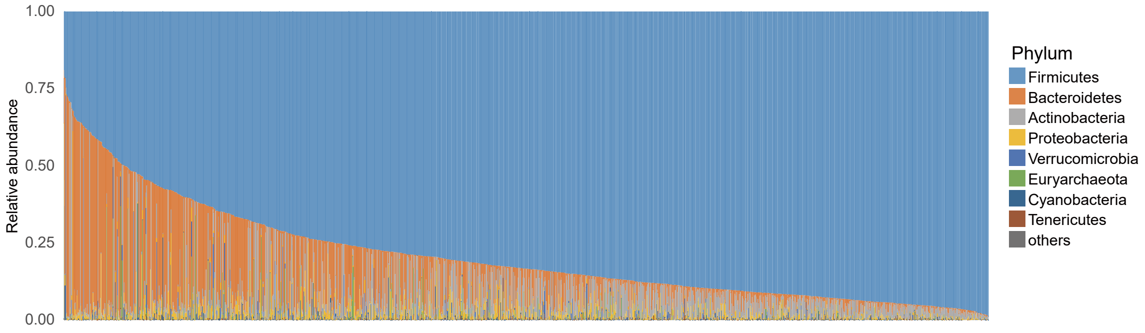

Supplementary Fig. 1. Composition of the pregnant women's gut microbiota at the phylum level.

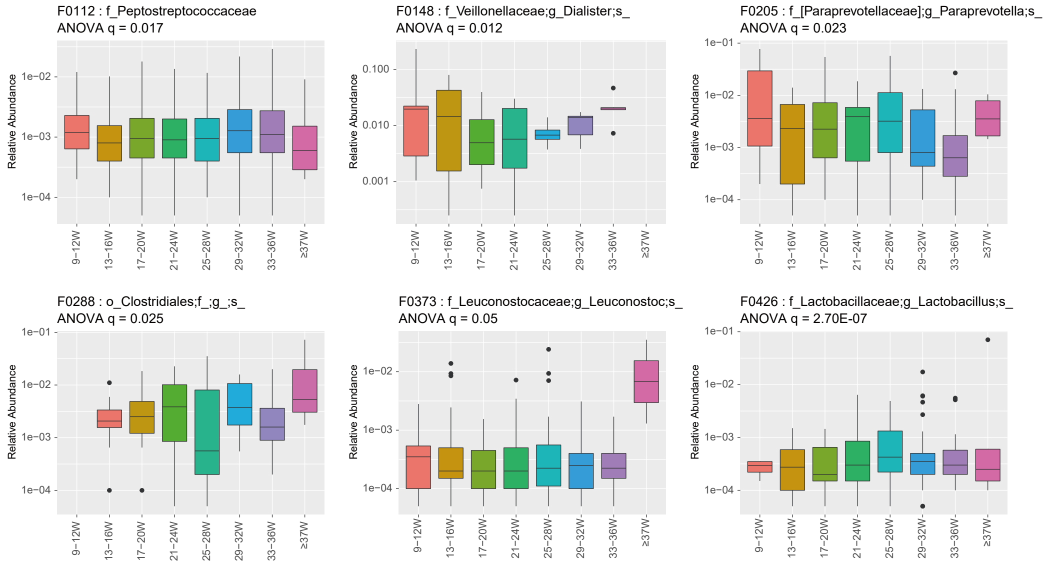

Supplementary Fig. 2. Distribution of OTUs that significant differed in relative abundance among the 8 gestational stages.

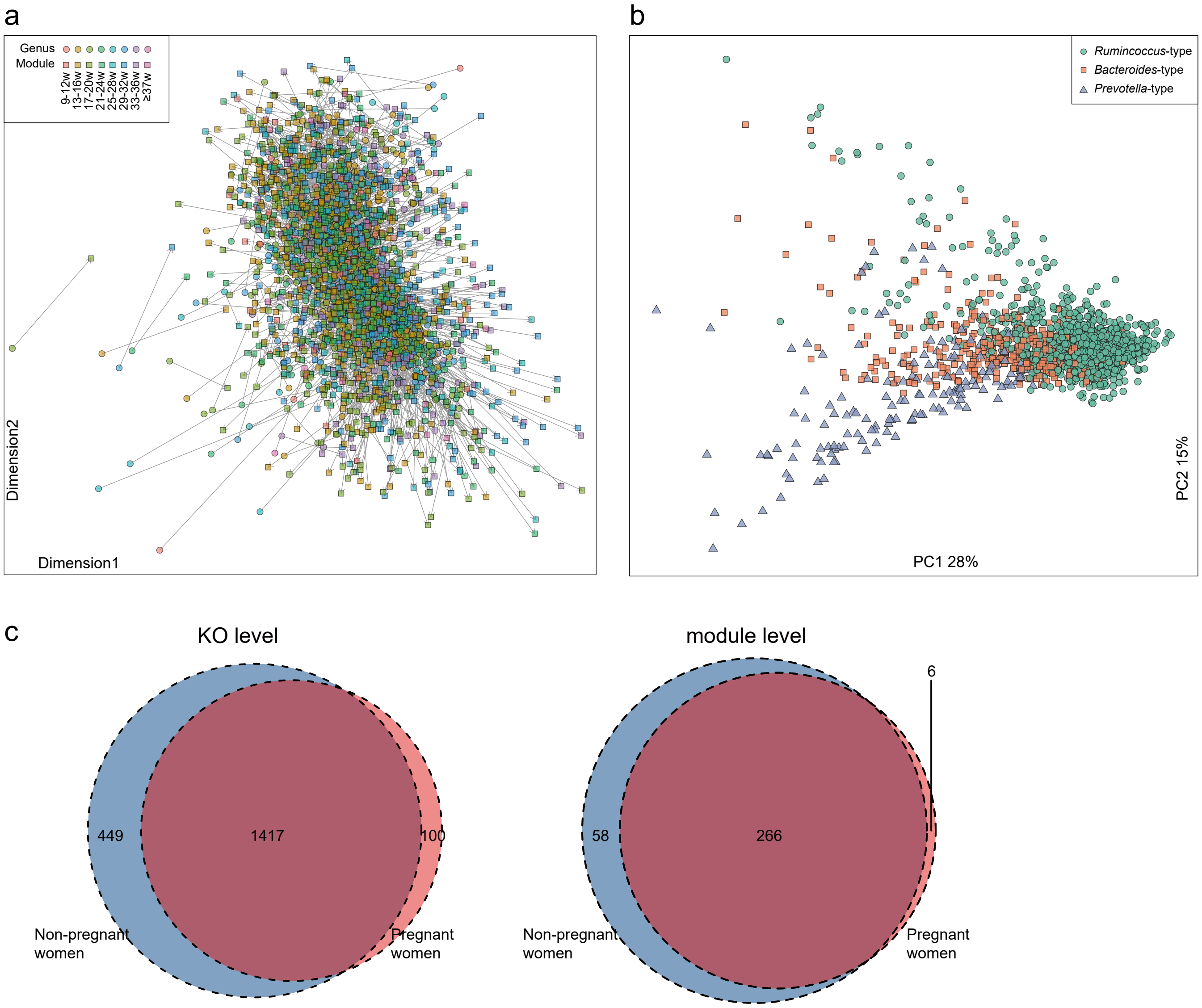

Supplementary Fig.3. The pregnant women's functional microbiome. **a** Procrustes analysis between microbial compositional and functional microbiomes. **b** Enterotype stratified the functional microbiome in pregnant women. Principal coordinates analysis (PCoA) based on the Bray-Curtis distance of KO profiles of pregnant women was shown. **c** Shared KO functional orthologs (left panel) and modules (right panel) between pregnant and nonpregnant women.

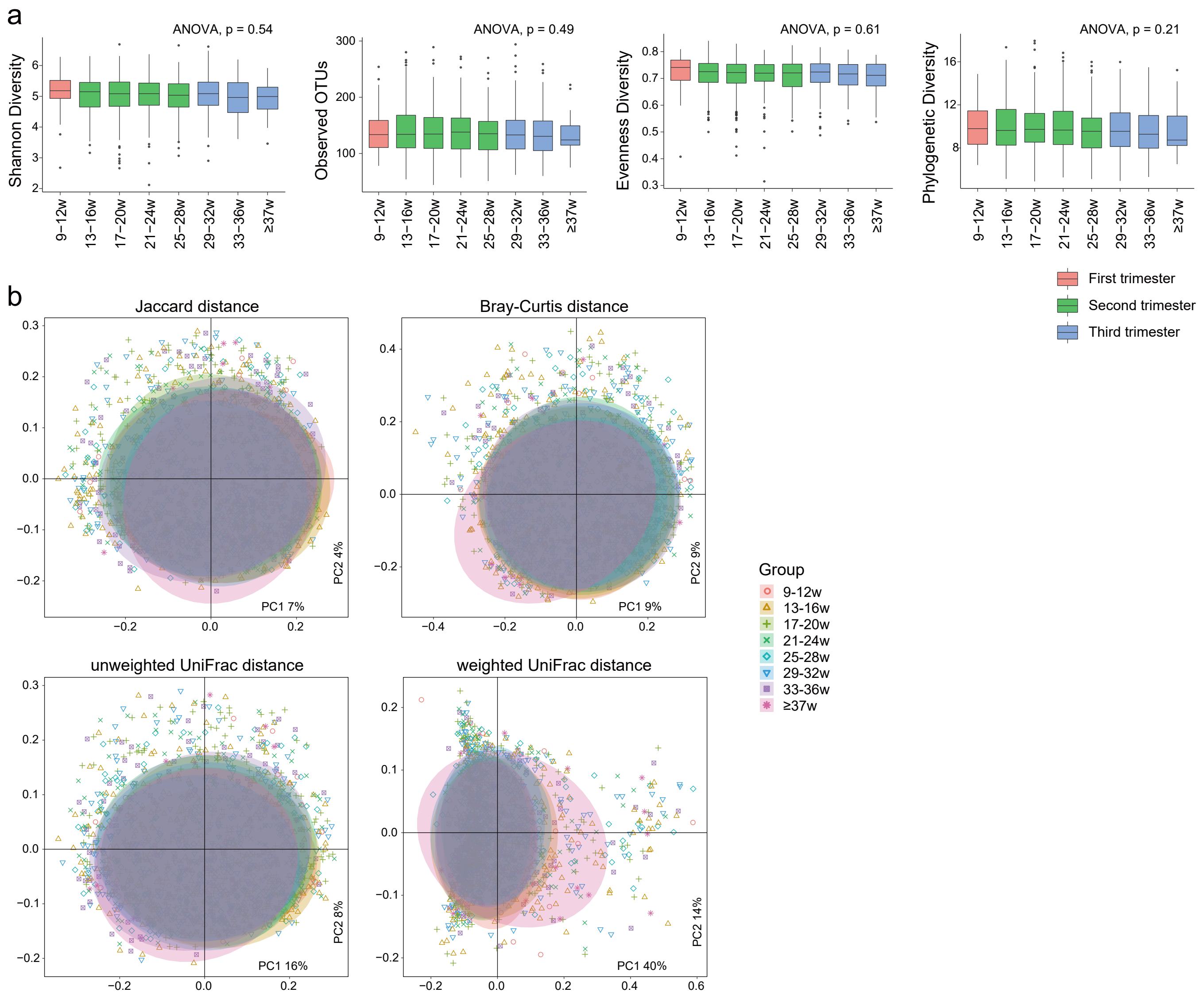

Supplementary Fig.4. Comparison of microbiota diversity among different gestational stages. **a** α-diversity comparison. The colors represented the first (red), second (green), and third (blue) trimesters of the samples of pregnant women. **b** PCoA analysis base on 4 β-diversity matrixes.

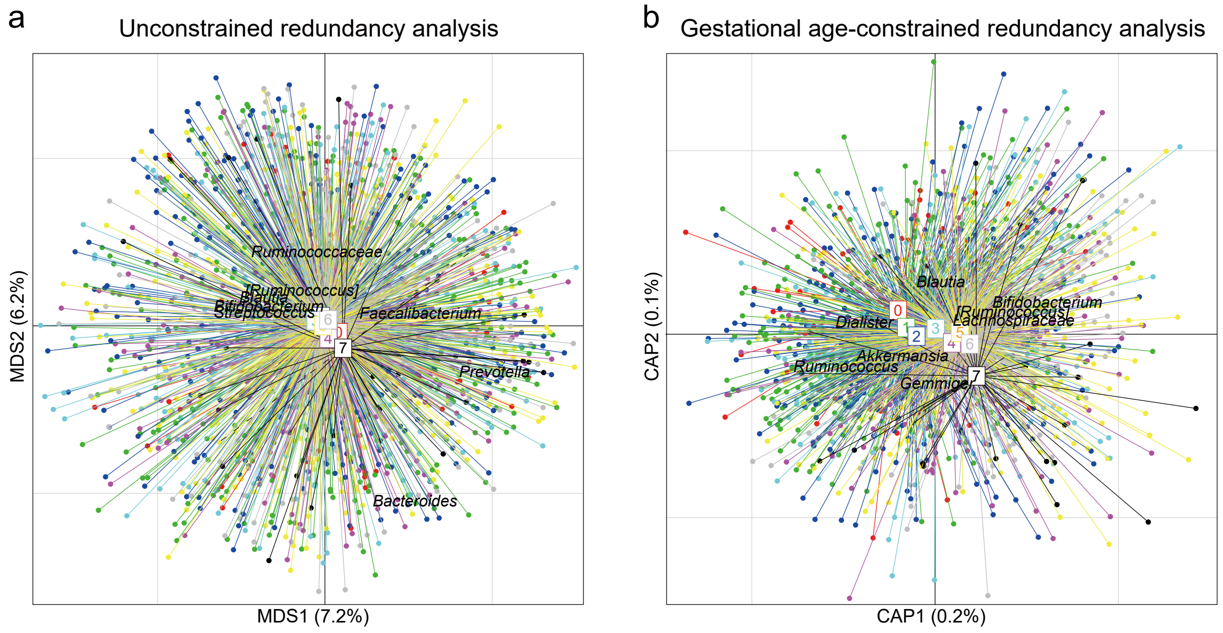

Supplementary Fig.5. Redundancy analysis of the gut microbiome of pregnant women. Unconstrained (**a**) and gestational-age constrained (**b**) redundancy analyses (RDA) was performed based on Bray-Curtis distance between microbial genera. Display of the unconstrained RDA was shown on sample scores on the primary and second multidimensional scales (MDS1 and MDS2), while display of the constrained RDA was shown on sample scores on the primary and second constrained axes (CAP1 and CAP2. Lines connected samples taken at the same gestational stages.

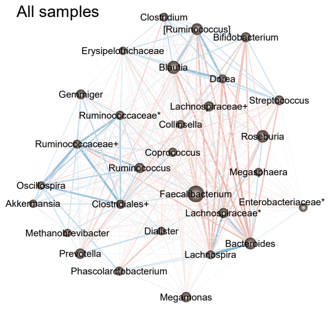

	# correlations	# shared with all samples	%
All	338	338	100.00%
9-12w	39	39	100.00%
13-16w	201	197	98.01%
17-20w	219	212	96.80%
21-24w	188	187	99.47%
25-28w	170	166	97.65%
29-32w	216	210	97.22%
33-36w	166	163	98.19%
⩾37w	14	14	100.00%

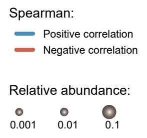

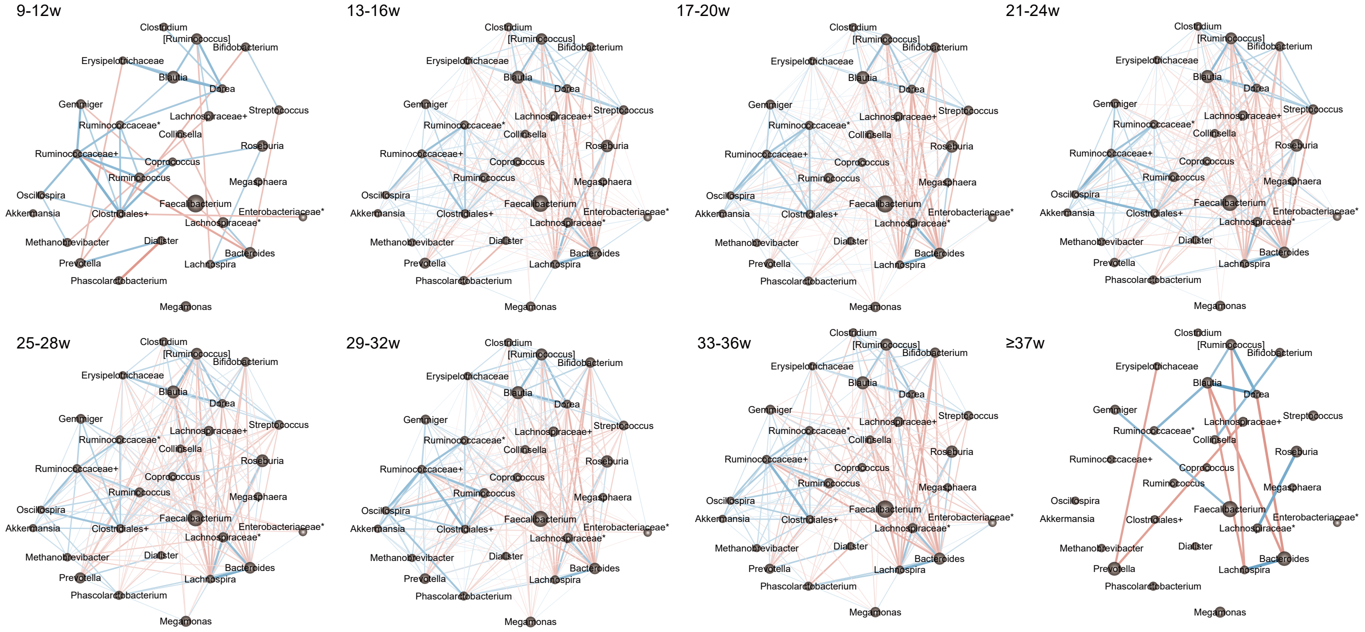

Supplementary Fig.6. Correlation network of the dynamics of 29 core genera. Correlation analysis was conducted on 29 core genera with average relative abundance >0.5% for all individuals or a subset of individuals at each gestational stage (Spearman's rank correlation coefficient test, $q \leq 0.05$). Blue line indicated [illegible]tive correlation, and red line indicated [illegible] correlation. Line color depth and width were proportional to degrees of the correlation. The table showed numbers of shared positive and negative correlation networks between genera for all individuals and individuals at each gestational stage.

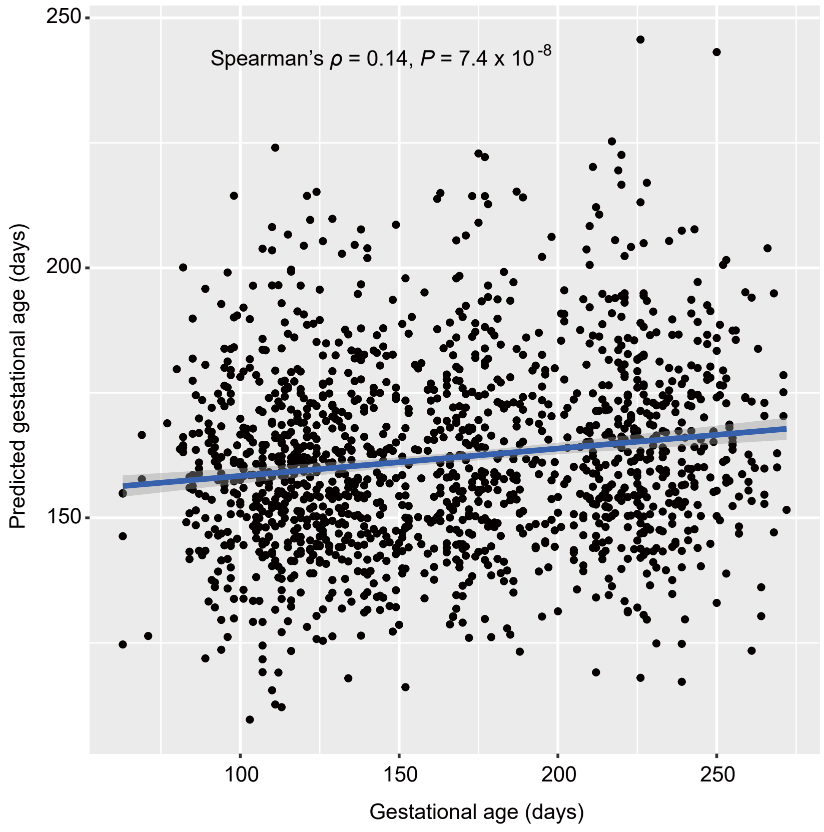

Supplementary Fig.7. Predicting the women's gestational age based on 11 gestational-age-associated genera.

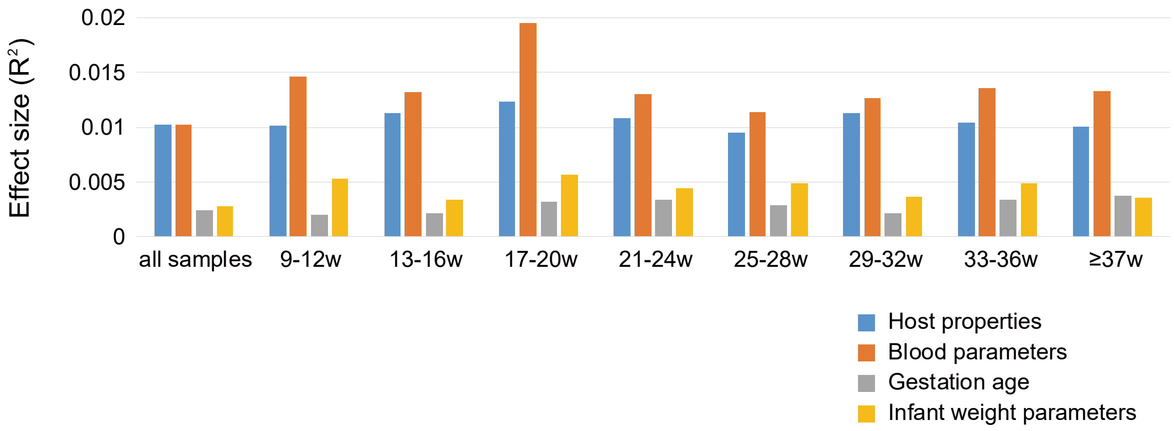

Supplementary Fig.8. The explicable variations of different categories of parameters for microbial community variation. Effect size was estimated using the function adonis with the default parameters in R.

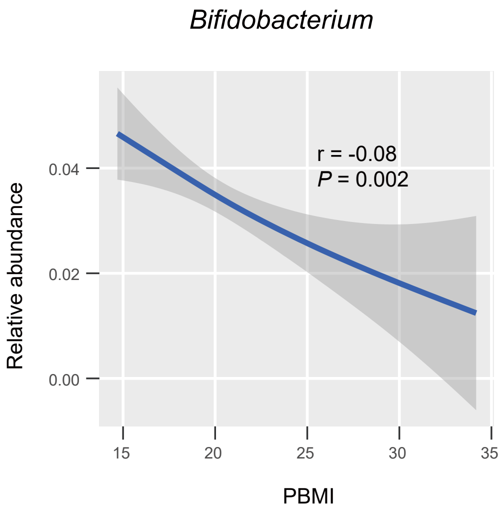

Supplementary Fig.9. Smooth curve based on the relative abundance of Bifidobacterium and pre-pregnancy body mass index (PBMI) of individuals. Smooth curve was formed using the function geom_smooth with the default parameters in R. The relationship and statistical significance between two data were determined by Spearman's rank correlation coefficient test.

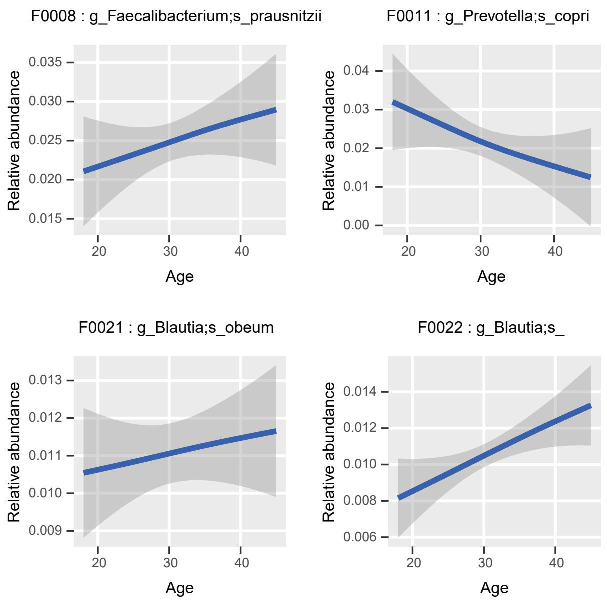

Supplementary Fig.10. Smooth curve based on the relative abundance of OTU and women's age. Smooth curve was formed using the function geom_smooth with the default parameters in R.

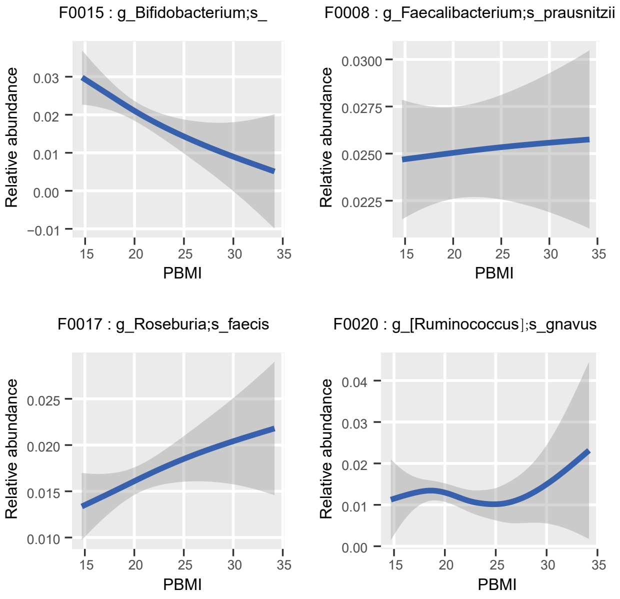

Supplementary Fig.11. Smooth curve based on the relative abundance of OTU and women's pre-pregnancy body mass index (PBMI). Smooth curve was formed using the function geom_smooth with the default parameters in R.

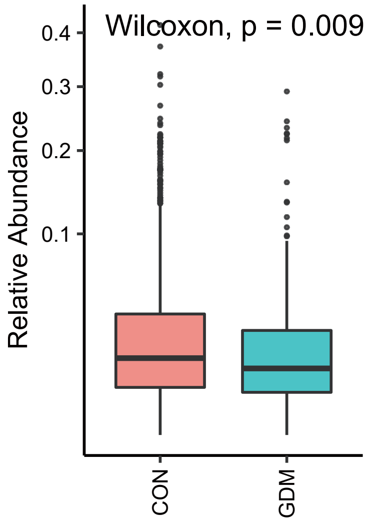

Supplementary Fig.12. The comparison of the relative abundance of genus Strepto-coccus between non-GDM and GDM women.

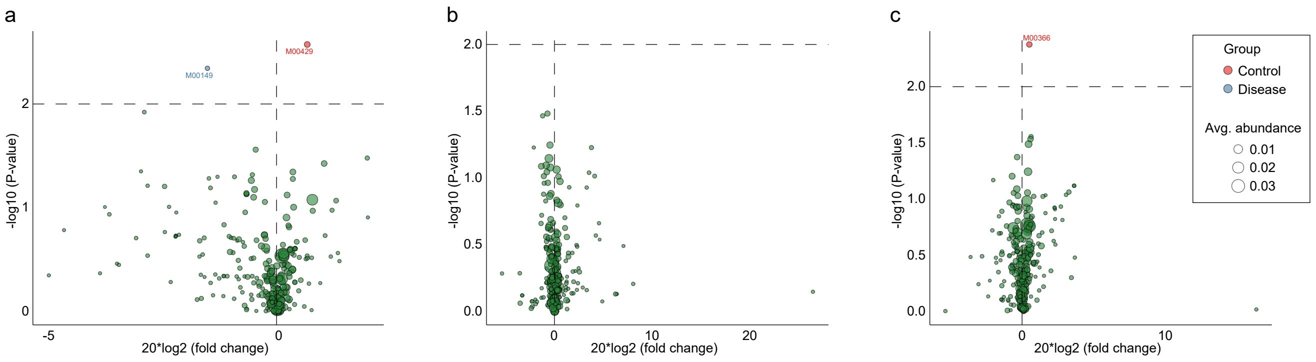

Supplementary Fig.13. The comparison of KEGG module-level D composition between control and disease group. **a** non-GDM versus GDM. **b** non-hepatopathy versus hepatopathy. **c** non-thalassemia versus thalassemia.
