# Supplementary material for: Systematic analysis of gut microbiota in pregnant women and its correlations with individual heterogeneity
Source: NPJ Biofilms Microbiomes. 2020 Sep 11;6:32. doi: 10.1038/s41522-020-00142-y (PMC7486914; doi:10.1038/s41522-020-00142-y)
Supplement: Supplementary file 2 — Description of Additional Supplementary Files [file 41522_2020_142_MOESM2_ESM.pdf]

Description of Additional Supplementary Files

File: Supplementary Data 1
Description: Summary of metadata.

File: Supplementary Data 2
Description: 29 core genera in the gut microbiota of pregnant women.

File: Supplementary Data 3
Description: 32 core genera in the gut microbiota of nonpregnant women.

File: Supplementary Data 4
Description: 489 core OTUs in the gut microbiota of pregnant women.

File: Supplementary Data 5
Description: 1,517 core KOs and 272 core modules.

File: Supplementary Data 6
Description: Adonis analysis of OTU, genus and KEGG.

File: Supplementary Data 7
Description: Correlation between host parameters and microbial genera.

File: Supplementary Data 8
Description: Age-associated OTUs and modules.

File: Supplementary Data 9
Description: PBMI-associated OTUs and modules.

File: Supplementary Data 10
Description: Resident type-associated OTUs and modules.

File: Supplementary Data 11
Description: GWG random forest regression using the core OTUs profile without added PBMI (left) and with added PBMI (right).

File: Supplementary Data 12
Description: GWG-associated OTUs and modules.

File: Supplementary Data 13
Description: The importance of OTUs to the accuracy of the model of random forest regression of GWG.

File: Supplementary Data 14
Description: Disease-associated OTUs and modules.

File: Supplementary Data 15
Description: Metadata of 1,479 participants of this study.

File: Supplementary Data 16
Description: The core OTUs profile of gut microbiota of pregnant women.

File: Supplementary Data 17
Description: The core OTUs profile of gut microbiota of nonpregnant women.
