# Supplementary material for: Systematic analysis of gut microbiota in pregnant women and its correlations with individual heterogeneity
Source: NPJ Biofilms Microbiomes. 2020 Sep 11;6:32. doi: 10.1038/s41522-020-00142-y (PMC7486914; doi:10.1038/s41522-020-00142-y)
Supplement: Supplementary file 20 — Reporting Summary [file 41522_2020_142_MOESM20_ESM.pdf]

nature research

Corresponding author(s): Shenghui Li

Last updated by author(s): Jul 24, 2020

Reporting Summary

Nature Research wishes to improve the reproducibility of the work that we publish. This form provides structure for consistency and transparency in reporting. For further information on Nature Research policies, see Authors & Referees and the Editorial Policy Checklist.

Statistics

For all statistical analyses, confirm that the following items are present in the figure legend, table legend, main text, or Methods section.

n/a	Confirmed	
☐	☒	The exact sample size (*n*) for each experimental group/condition, given as a discrete number and unit of measurement
☐	☒	A statement on whether measurements were taken from distinct samples or whether the same sample was measured repeatedly
☐	☒	The statistical test(s) used AND whether they are one- or two-sided<br>*Only common tests should be described solely by name; describe more complex techniques in the Methods section.*
☐	☒	A description of all covariates tested
☐	☒	A description of any assumptions or corrections, such as tests of normality and adjustment for multiple comparisons
☐	☒	A full description of the statistical parameters including central tendency (e.g. means) or other basic estimates (e.g. regression coefficient) AND variation (e.g. standard deviation) or associated estimates of uncertainty (e.g. confidence intervals)
☐	☒	For null hypothesis testing, the test statistic (e.g. *F*, *t*, *r*) with confidence intervals, effect sizes, degrees of freedom and *P* value noted<br>*Give P values as exact values whenever suitable.*
☒	☐	For Bayesian analysis, information on the choice of priors and Markov chain Monte Carlo settings
☒	☐	For hierarchical and complex designs, identification of the appropriate level for tests and full reporting of outcomes
☐	☒	Estimates of effect sizes (e.g. Cohen's *d*, Pearson's *r*), indicating how they were calculated

Our web collection on statistics for biologists contains articles on many of the points above.

Software and code

Policy information about availability of computer code

Data collection	https://github.com/RChGO/PregnantMicrobiota ; European Nucleotide Archive (https://www.ebi.ac.uk/ena)
Data analysis	R v3.6.0; Qiime2 v 2018.6.0

For manuscripts utilizing custom algorithms or software that are central to the research but not yet described in published literature, software must be made available to editors/reviewers. We strongly encourage code deposition in a community repository (e.g. GitHub). See the Nature Research guidelines for submitting code & software for further information.

Data

Policy information about availability of data

All manuscripts must include a data availability statement. This statement should provide the following information, where applicable:

- Accession codes, unique identifiers, or web links for publicly available datasets
- A list of figures that have associated raw data
- A description of any restrictions on data availability

The raw sequencing dataset analyzed in this study has been deposited in the European Bioinformatics Institute (EBI) database under the accession code PRJEB31743. The sample metadata was provided as Supplementary Data for reproducibility of this study.

Field-specific reporting

Please select the one below that is the best fit for your research. If you are not sure, read the appropriate sections before making your selection.

☒ Life sciences ☐ Behavioural & social sciences ☐ Ecological, evolutionary & environmental sciences

October 2018

For a reference copy of the document with all sections, see nature.com/documents/nr-reporting-summary-flat.pdf

Life sciences study design

All studies must disclose on these points even when the disclosure is negative.

Sample size	Sample-size calculation was not performed. We collected as many samples as possible to make our analysis more representative for gut microbiota in pregnant women.
Data exclusions	Faecal samples from pregnant women who do not match the exclusion can be used for our analysis . Our exclusion criteria included 1) usage of the following drugs in the previous 6 months: systemic antibiotics, corticosterone, cytokines, methotrexate or other immunotoxic drugs, hormonal contraceptives, and high dose of commercial probiotics; 2) presence of risky diseases: serious cardiovascular disease, inflammatory bowel disease, irritable bowel syndrome, and celiac disease; 3) human immunodeficiency virus infection; 4) intestinal surgery within 5 years; 5) chronic diarrhea caused by Clostridium difficile or an unknown agent; 6) chronic constipation; 7) unusual dietary habits such as individuals with alcoholism and strict vegetarians; and 8) conventional antibiotic treatment or probiotic supplement in the preceding four weeks.
Replication	The study was a large-scale cohort and we didn't try to replicate all aspects of sample collection and data generation. However, datasets and custom code about the study are available in GitHub and our analysis were performed based on public and widely used methods (details in Methods), which ensure that our results can be repeated well.
Randomization	All pregnant women were allocated into groups based on medical information. Each grouping criteria and sample number in groups have been shown in Supplementary Data 1.
Blinding	Faecal samples of all pregnant women who volunteered to join the study were collected in Guangzhou Women and Children's Medical Center and investigators retained samples that do not match the exclusion mentioned above for sequencing. All comparative analysis was performed based on individual clinical information.

Reporting for specific materials, systems and methods

We require information from authors about some types of materials, experimental systems and methods used in many studies. Here, indicate whether each material, system or method listed is relevant to your study. If you are not sure if a list item applies to your research, read the appropriate section before selecting a response.

Materials & experimental systems

n/a	Involved in the study	
☒	☐	Antibodies
☒	☐	Eukaryotic cell lines
☒	☐	Palaeontology
☒	☐	Animals and other organisms
☒	☐	Human research participants
☒	☐	Clinical data

Methods

n/a	Involved in the study	
☒	☐	ChIP-seq
☒	☐	Flow cytometry
☒	☐	MRI-based neuroimaging
